# Elucidation of the O-antigen structure of *Escherichia coli* O93 and characterization of its biosynthetic genes

**DOI:** 10.1093/glycob/cwac069

**Published:** 2022-10-14

**Authors:** Axel Furevi, Jonas Ståhle, Claudio Muheim, Spyridon Gkotzis, Daniel O Daley, Klas I Udekwu, Göran Widmalm

**Affiliations:** Department of Organic Chemistry, Arrhenius Laboratory, Stockholm University, S-106 91 Stockholm, Sweden; Department of Organic Chemistry, Arrhenius Laboratory, Stockholm University, S-106 91 Stockholm, Sweden; Department of Biochemistry and Biophysics, Arrhenius Laboratory, Stockholm University, S-106 91 Stockholm, Sweden; Department of Molecular Biosciences, The Wenner-Gren Institute, Stockholm University, S-106 91 Stockholm, Sweden; Department of Biochemistry and Biophysics, Arrhenius Laboratory, Stockholm University, S-106 91 Stockholm, Sweden; Department of Molecular Biosciences, The Wenner-Gren Institute, Stockholm University, S-106 91 Stockholm, Sweden; Department of Aquatic Sciences and Assessment, Swedish University of Agriculture, PO Box 7050, SE-750 07 Uppsala, Sweden; Department of Organic Chemistry, Arrhenius Laboratory, Stockholm University, S-106 91 Stockholm, Sweden

**Keywords:** bioinformatics, CarbBuilder, CASPER, lipopolysaccharide, NMR spectroscopy

## Abstract

The structure of the O-antigen from the international reference strain *Escherichia coli* O93:−:H16 has been determined. A nonrandom modal chain-length distribution was observed for the lipopolysaccharide, a pattern which is typical when long O-specific polysaccharides are expressed. By a combination of (i) bioinformatics information on the gene cluster related to O-antigen synthesis including putative function on glycosyl transferases, (ii) the magnitude of NMR coupling constants of anomeric protons, and (iii) unassigned 2D ^1^H, ^13^C-HSQC, and ^1^H,^1^H-TOCSY NMR spectra it was possible to efficiently elucidate the structure of the carbohydrate polymer in an automated fashion using the computer program CASPER. The polysaccharide also carries *O*-acetyl groups and their locations were determined by 2D NMR experiments showing that ~½ of the population was 2,6-di-*O*-acetylated, ~¼ was 2-*O*-acetylated, whereas ~¼ did not carry *O*-acetyl group(s) in the 3-*O*-substituted mannosyl residue of the repeating unit. The structure of the tetrasaccharide repeating unit of the O-antigen is given by: →2)-β-d-Man*p*-(1→3)-β-d-Man*p*2Ac6Ac-(1→4)-β-d-Glc*p*A-(1→3)-α-d-Glc*p*NAc-(1→, which should also be the biological repeating unit and it shares structural elements with capsular polysaccharides from *E. coli* K84 and K50. The structure of the acidic O-specific polysaccharide from *Cellulophaga baltica* strain NN015840^T^ differs to that of the O-antigen from *E. coli* O93 by lacking the *O*-acetyl group at O6 of the *O*-acetylated mannosyl residue.

## Introduction

*Escherichia coli* is a bacterium that belongs to the family *Enterobacteriaceae* and it is part of the colonic flora of humans, where the bacterium and the host mutually benefit from each other. However, clinical syndromes that result from pathogenic strains include enteric/diarrheal disease, urinary tract infection, and sepsis/meningitides (Stenutz et al. 2006; Vila et al. 2016; Liu et al. 2020).

Some *E. coli* make Shiga toxin(s) known as Stx1, one of the most potent bacterial toxins described to date (Melton-Celsa 2014), though a second type referred to as Stx2 is present in certain *E. coli* strains. Collectively these bacteria are referred to as Shiga toxin producing *E. coli* (STEC) and this pathogen is a potential health risk as it may cause, in particular, hemolytic uremic syndrome (HUS) resulting in kidney failure. The prevalence of STEC in healthy adults in Japan was investigated by collecting close to 3 million samples during a 2-year period and a large number of serogroups was identified carrying *stx* genes, among others, *E. coli* O93 (Morita-Ishihara et al. 2016). Monitoring of food-producing animals during handling and preparation of meat is therefore important in order to avoid STEC-based outbreaks and illness caused by these bacteria. The use of venison for human consumption called for a closer investigation of potential contamination by STEC, e.g. from wild deer in Japan where 4 serogroups were isolated, one of them being *E. coli* O93 (Asakura et al. 1998). In a more recent study of 30 sika deer in Japan during 3 years the most dominant serogroup was O93 present in almost 40% of the isolated samples (Kabeya et al. 2017). In bovine ground meat from Brazil, *E. coli* O93 was not only identified as STEC but also as an enteroaggregative *E. coli* (EAEC) pathotype (Pelayo et al. 2019). Furthermore, bacterial *E. coli* isolates from cattle in Australia revealed that serogroup O93 was common and it was shown to have an antibiotic resistance phenotype, though the specific antibiotics to which the *E. coli* O93 bacteria were resistant did vary (Bettelheim et al. 2005). Avian pathogenic *E. coli* (APEC) were isolated from ducks in China during an 8-year period, where *E. coli* O93 was the predominant serogroup (Wang et al. 2010), whereas in ducks from Vietnam it was the least common of the isolated serogroups (Thu et al. 2019). A study of virulence factors in *E. coli* isolates from bovine mastitis (Fernandes et al. 2011) identified serogroups in a few cases, one of them being *E. coli* O93, where also the somatic antigen was possible to identify, i.e. the O-antigen. Furthermore, this *E. coli* O93 isolate was resistant to the antibiotics ampicillin and trimethoprim sulfa.

The potential severity as a human pathogen has prompted us to investigate the structure of the O-antigen polysaccharide of *E. coli* O93 as knowledge thereof may give insight to how the bacterium can evade the immune system of the host, and it may also form the basis for future vaccine candidates. To this end we have used a combination of bioinformatics information, NMR spectroscopy and the structure prediction tool CASPER to unravel its primary structure. Subsequently, 3-dimensional sub-structure models of the polysaccharide were possible to construct revealing polymer extension and epitope accessibility of structural entities.

## Results and discussion

**Fig. 1:**
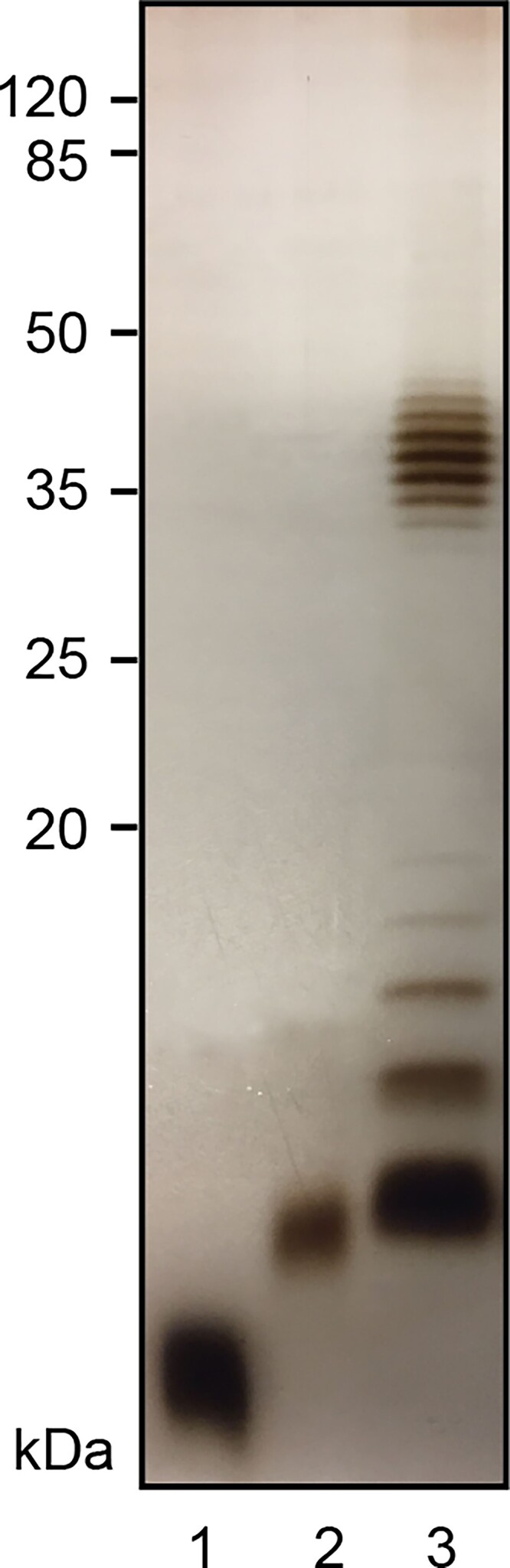
SDS-PAGE analysis of LPS from different *E. coli* strains. Lane 1: LPS from *E. coli* CWG303 Δ*waaG*, Lane 2: LPS from *E. coli* W3110, and Lane 3: LPS from *E. coli* O93. A 14% Tricine SDS-PAGE gel was used to separate LPS samples, which subsequently were stained with AgNO_3_. A pre-stained protein marker (Thermo Fisher Scientific) was utilized for calibration of molecular weights.

*Escherichia coli* O93 was grown in a lysogeny broth medium and the LPS was extracted according to the hot phenol/water method using previously described procedures (Furevi et al. 2020). The LPS was analyzed by SDS-PAGE (Fig. 1, lane 3). This analysis revealed a typical ladder pattern for the LPS from *E. coli* O93 owing to the O-antigen having a number of repeating units (RU) expressed with a nonrandom modal chain-length distribution. For comparison, the rough LPS from *E. coli* CWG303 Δ*waaG* (lane 1; Yethon et al. 2000) and *E. coli* W3110 (lane 2; Klein et al. 2013) were included. *Escherichia coli* CWG303 ΔwaaG is truncated at the heptose region of the inner core whereas *E. coli* W3110 contains the full K12 core. An initial analysis by ^1^H NMR spectroscopy of the LPS revealed the presence of signals in the spectral region 2.00–2.05 and 2.17–2.22 ppm where resonances from methyl groups of *N*-acetyl and *O*-acetyl groups of sugar residues reside, respectively.

**Fig. 2:**
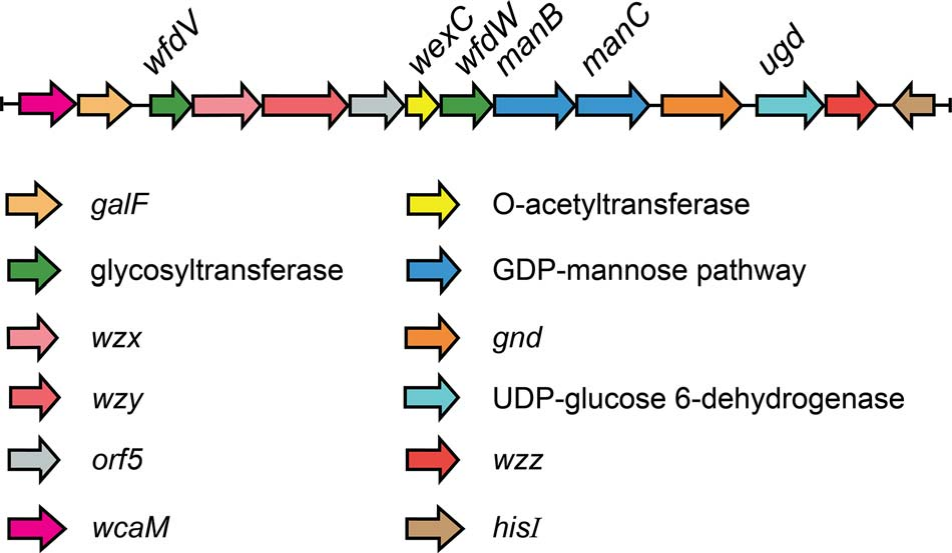
The O-antigen gene cluster between *galF* and *hisI* in *E. coli* O93 (Iguchi et al. 2015; DebRoy et al. 2016; Liu et al. 2020).

The part of the genome of *E. coli* O93 related to O-antigen synthesis and expression, from *galF* to *HisI*, has been deposited at NCBI (GenBank AB812041.1), analyzed and putative functions of the gene products have been proposed and annotated (Fig. 2; Iguchi et al. 2015; DebRoy et al. 2016; Liu et al. 2020) The presence of the genes *wzx*, *wzy*, and *wzz* indicates that the O-antigen is synthesized via the Wzx/Wzy biosynthetic pathway (Reeves et al. 1996; Islam and Lam 2014; Liu et al. 2019; Ståhle and Widmalm 2019; Whitfield et al. 2020). The gene cluster also contained 2 NDP-sugar precursor-encoding genes, *manB* and *manC*, involved in the synthesis of the donor GDP-d-Man*p*, having an α-anomeric configuration. Furthermore, a putative *O*-acetyl transferase, *wexC*, was proposed and by identification of conserved regions it can be shown to be maltose O-acyltransferase (MAT)-like (Lo Leggio et al. 2003). A gene annotated as *ugd* was also present. This gene most likely encodes a UDP-glucose 6-dehydrogenase that converts UDP-glucose to UDP-glucuronic acid (Stevenson et al. 1996) (UDP-d-Glc*p*A with an α-anomeric configuration). Two genes coding for glycosyltransferases (GTs) were identified, viz., *wfdV* and *wfdW*, after *galF* and *wexC*, respectively. Analysis and identification of conserved regions revealed that *wfdV* belongs to GT family 2 that acts through an inverting mechanism whereas a corresponding analysis showed that *wfdW* belongs to GT family 1, which also acts through an inverting mechanism. The function of the product coded by the gene denoted by *orf5* has not yet been identified.

The Wzx/Wzy-dependent pathway employs WecA to make Und-*PP*-d-GlcNAc (Al-Dabbagh et al. 2016) as the initial sugar of the biological repeating unit (BRU). Since both *gnu*, coding an epimerase that interconverts *N*-acetyl-d-glucosamine-*PP*-Und to *N*-acetyl-d-galactosamine-*PP*-Und, and *gne*, coding for an epimerase that isomerases UDP-d-Glc*p*NAc to UDP-d-Gal*p*NAc (Cunneen et al. 2013), are absent in the genome as judged from analysis hitherto performed, we conclude that the first sugar residue of the BRU is d-Glc*p*NAc and that d-Gal*p*NAc is not present in the O-antigen (unless *orf5* corresponds to one of the two genes). As there are not any other genes in the sequence between *galF* and *gnd* the sugar residues in the RU should contain d-GlcNAc and d-Man as well as maybe d-GlcA; sugar residues coded outside the O-antigen cluster by housekeeping genes, e.g. d-Glc and d-Gal, may also be constituents of the O-polysaccharide.

Databases storing information on polysaccharide structures facilitate comparison among entries, which can be highly beneficial when unknown structures are to be elucidated. ECODAB stores (for each serogroup) available information from literature on *E. coli* O-antigen structures, their NMR chemical shifts and function of GTs used in the biosynthesis of the O-polysaccharides (Lundborg et al. 2010; Rojas-Macias 2015). Importantly, local BLAST+ searches have been performed within the database to compare amino acid sequences of putative GTs and the results from the sequence similarity searches are stored and ranked according to the e-value (the lower the value the more significant is the agreement) based on the similarity to other GTs within the database, for which function is known or has been predicted. In this way structural information can be gained resulting in a disaccharide structural element whose two constituent monosaccharides, the anomeric configuration at the glycosidic linkage and the substitution position of the acceptor sugar can be proposed. For *E. coli* O93 O-antigen biosynthesis, the first gene in the sequence coding for a putative GT, *wfdV*, is closely similar, with an e-value of 3 × 10^−101^, to *weiG* from *E. coli* O161 (Li et al. 2010). *weiG* codes for a GT that makes the disaccharide structural element β-d-Glc*p*A-(1→3)-d-Glc*p*NAc, which is consistent with the fact that WfdV is proposed to operate via an inverting mechanism and that UDP-d-Glc*p*A with an α-anomeric configuration should then be the donor molecule as the product has the β-anomeric configuration.

**Fig. 3:**
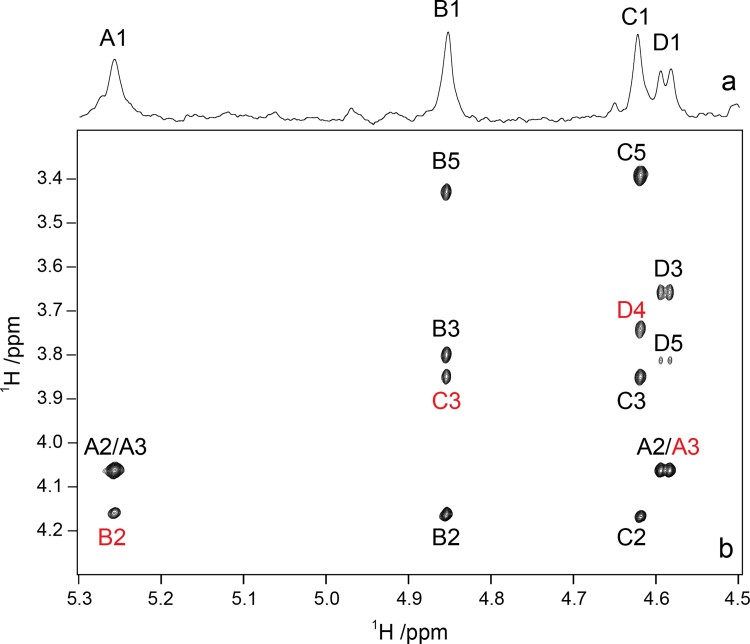
The anomeric region of *O*-deacetylated *E. coli* O93 O-antigen polysaccharide (PS–OH) from a ^1^H NMR spectrum (a) and a ^1^H,^1^H-NOESY spectrum with a mixing time of 150 ms at 700 MHz (b). Transglycosidic cross-peaks are annotated by red color.

Polysaccharide material was prepared by treatment of the LPS with dilute acid, dialysis, and gel-permeation chromatography as well as by treatment under dilute basic conditions to yield material referred to as PS and PS–OH, respectively. The ^1^H NMR spectrum of the PS–OH sample showed four resonances from anomeric protons and the sugar residues corresponding to these were denoted **A–D** by order of descending proton chemical shift (Fig. 3a). Notably, the ^3^*J*_H1,H2_ of the anomeric protons of residues **B** and **C** were small and estimated to have ^3^*J*_H1,H2_ < 2 Hz, which is characteristic for *manno*-configured monosaccharides. The anomeric proton of **D** showed ^3^*J*_H1,H2_ of ~8 Hz whereas H1 of **C** was significantly broader in comparison to those from **B** and **C** and was therefore estimated to have a medium-sized ^3^*J*_H1,H2_ ≈ 3–4 Hz, which is characteristic for pyranose sugar residues having the *gluco*/*galacto*-configuration.

**Fig. 4:**
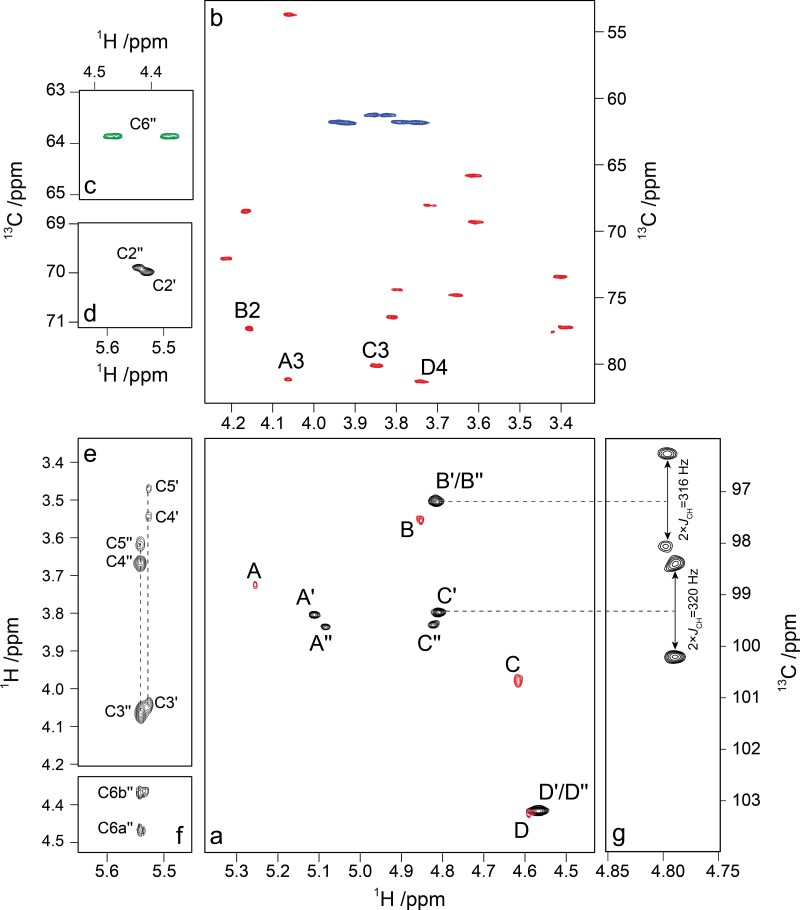
NMR spectra of selected regions from the O-antigen polysaccharides (PS and PS–OH) of *E. coli* O93. (a) An overlay of ^1^H,^13^C-meHSQC NMR spectra of the anomeric region from PS (black) and PS–OH (red); (b) ^1^H,^13^C-meHSQC spectrum of PS–OH plotted in red color with linkage positions annotated and hydroxymethyl correlations plotted in blue color; (c, d) selected regions from the ^1^H,^13^C-meHSQC spectrum of the PS sample showing the *O*-acetylated positions in residues **C′**/**C″** with hydroxymethyl correlations plotted in green color; (e, f) ^1^H,^1^H-TOCSY spectrum with a mixing time of 120 ms displaying the cross-peaks from H2 resonances in residues **C2′** and **C2″**; (g) selected region from the coupling-enhanced (2×) ^1^H,^13^C-CT-CE-HSQC NMR spectrum resulting in ^1^*J*_C1,H1_ for **B′/B″** (158 Hz) and **C′/C″** (160 Hz).

A ^1^H,^13^C-meHSQC (multiplicity-edited) NMR spectrum of the O93 PS–OH showed a total of 27 cross-peaks consisting of 4 cross-peaks in the spectral region for anomeric atoms (Fig. 4a), 15 cross-peaks with ^13^C chemical shifts in the spectral range 65–82 ppm, 3 pairs of hydroxymethyl related resonances at *δ*_C_ 60–64, one cross-peak from a nitrogen-bearing carbon at *δ*_C_ 53.70 (Fig. 4b) and a resonance at *δ*_H_/*δ*_C_ 2.04/22.87 in the *N*-acetyl spectral region. The bioinformatics analysis suggested 3 different sugar residues and together with the NMR data we conclude that the fourth sugar residue in the O-antigen should be a hexose. Furthermore, the ^1^H NMR data of the resonances from anomeric protons are consistent with the fact that two mannosyl residues should be present as part of the RU of the O-antigen polysaccharide. That the absolute configuration is d for the sugar residues in the O-antigen polysaccharide is deduced based on biosynthetic considerations. A sugar analysis of the polysaccharide showing that the major hexose component was mannose supports the above conclusion based on the bioinformatics analysis. In addition, a minor hexose component was detected by the sugar analysis, viz., glucose, and it is assumed to derive from the core region of the LPS.

To determine the structure of the PS–OH, spectral data from a 1D ^1^H NMR spectrum and the magnitude of ^3^*J*_H1,H2_ coupling constants, 2D ^1^H,^13^C-meHSQC and *F*_2_-coupled ^1^H,^13^C-meHSQC spectra where the latter reveals the magnitude of ^1^*J*_C1,H1_ coupling constants (1 with ^1^*J*_C1,H1_ > 170 Hz and 3 with ^1^*J*_C1,H1_ ≈ 160 Hz), and correlations from ^1^H,^1^H-TOCSY NMR spectra were used. The computer program CASPER can determine polysaccharide structure using NMR data in conjunction with some additional information such as sugar components (Jansson et al. 1987b; Lundborg and Widmalm 2011). The information obtained from the bioinformatics analysis and the sugar analysis, including the fact that the O-antigen is supposed to be synthesized via the Wzx/Wzy-dependent pathway (referred to as the biological WecA rule in CASPER), was given as input to CASPER, i.e. the sugar residues d-GlcNAc, d-Glc*p*A, and d-Man twice as well as the structural element β-d-Glc*p*A-(1→3)-d-Glc*p*NAc in conjunction with the above described *unassigned* NMR data (Supporting information).

**Fig. 5:**
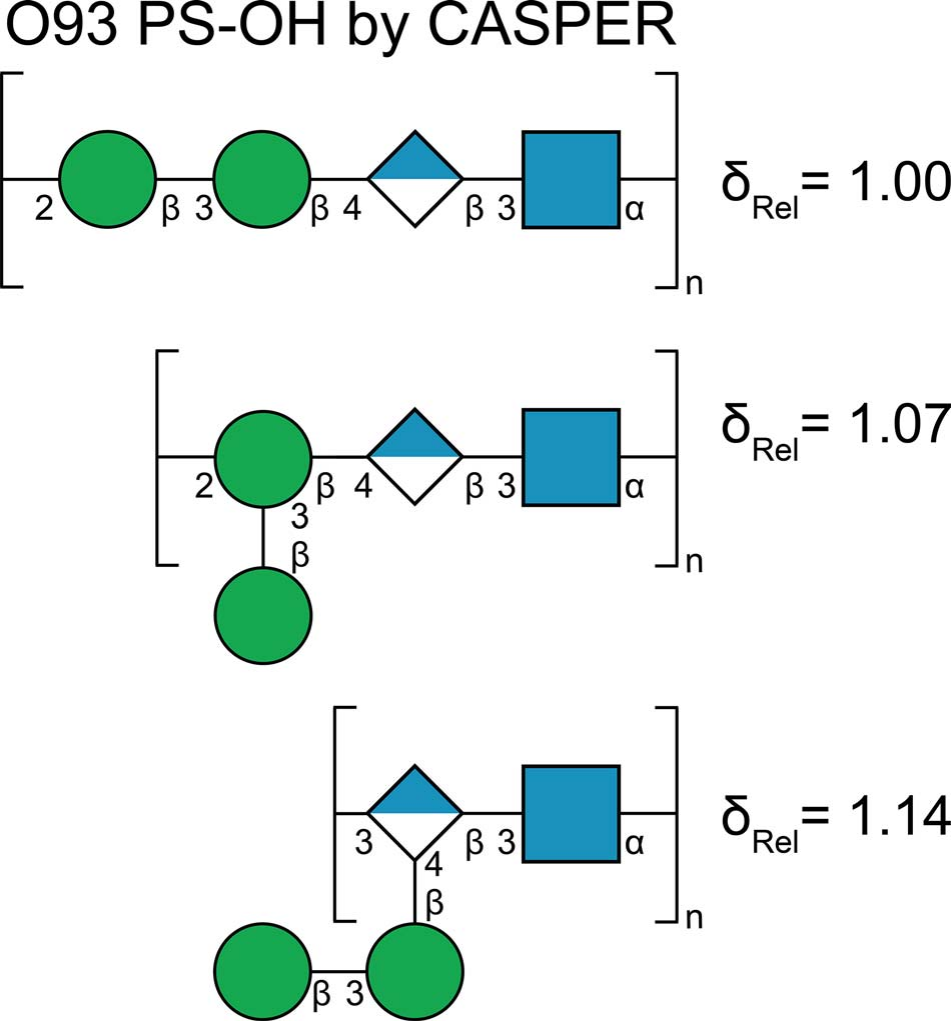
Top 3 structures using the “Determine Glycan Structure” entry in CASPER for *E. coli* O93 PS–OH with NMR data obtained from ^1^H,^13^C-HSQC and ^1^H,^1^H-TOCSY experiments and magnitudes of coupling constants for input to CASPER, viz., those from ^1^*J*_C1,H1_ and ^3^*J*_H1,H2_ obtained from coupled ^1^H,^13^C-HSQC and ^1^H NMR spectra, respectively.

The highest ranked O93 PS–OH structure proposed by CASPER has a linear tetrasaccharide RU in which both of the mannose residues have the β-anomeric configuration (Fig. 5), whereas the second and third ranked structures both have branched RUs. The proposed structure was confirmed by NMR resonance assignments of the PS–OH. The four sugar residues should have the pyranoid ring-form as indicated from ^13^C NMR chemical shift data (Jansson et al. 1989, 1991) and that characteristic signals for furanosides (Ritchie et al. 1975; Beier et al. 1980) were absent in the ^1^H,^13^C-meHSQC spectrum. Residue **A** having ^1^*J*_C1,H1_ > 170 Hz shows that it has the α-anomeric configuration, whereas residues **B**–**D** have ^1^*J*_C1,H1_ ≈ 160 Hz revealing that they all have the β-anomeric configuration (Table 1; Bundle and Lemieux 1976).

**Fig. 6:**
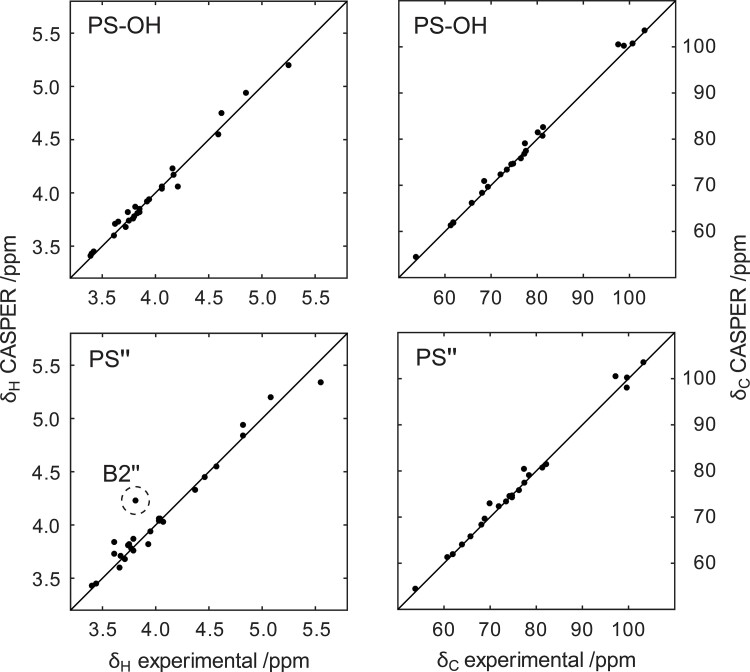
Comparison of ^1^H (left) and ^13^C (right) NMR chemical shifts predicted by CASPER versus assigned from NMR experiments for the *O*-deacetylated (PS–OH) and the 2,6-di-*O*-acetylated population of the polysaccharide (PS) from the *E. coli* O93 O-antigen.

Residue **A** whose anomeric proton resonates at *δ*_H_ 5.25 showed at a short mixing a correlation to *δ*_H_ 4.06 in the ^1^H,^1^H-TOCSY spectrum and the latter correlated to the single ^13^C resonance at *δ*_C_ 53.70 in the ^1^H,^13^C-meHSQC NMR spectrum; this sugar residue was therefore assigned to α-d-Glc*p*NAc. At longer mixing times the intensity of the cross-peak increased further and it was not until long mixing times were used that the full spin-system could be identified (Table 1). Thus, also H3 resonates at *δ*_H_ 4.06 and correlates to a glycosyloxylated carbon-13 nucleus resonating at 81.15 ppm, consistent with a large downfield chemical shift displacement, Δ*δ*_C3_ 9.41, due to substitution at O3 in →3)-α-d-GlcNAc-(1→. The *manno*-configured sugars **B** and **C** had spin-systems that showed correlations between their anomeric protons and their H2 protons at *δ*_H_ 4.16 and *δ*_H_ 4.17, respectively, only when the longest mixing time of 120 ms was employed in the ^1^H,^1^H-TOCSY experiments. The remaining part of their spin-systems were assigned in a stepwise manner from the H2 autopeak using ^1^H,^1^H-TOCSY NMR spectra with incremented mixing times. In addition, strong intra-residue correlations were observed from H1 to H2, H3, and H5 in the ^1^H,^1^H-NOESY spectrum (Fig. 3b), which further confirms that both sugar residues have the β-anomeric configuration. The glycosylation shifts of residues **B** and **C**, Δ*δ*_C2_ 5.20 and Δ*δ*_C3_ 6.08, reveal that they correspond to →2)-β-d-Man-(1→ and →3)-β-d-Man-(1→, respectively. ^1^H,^1^H-TOCSY NMR spectra with increasing duration of the spin-lock times were used to assign ^1^H NMR chemical shifts of residue **D**, the anomeric proton of which resides at *δ*_H_ 4.59, up to H5 having a chemical shift of 3.81 ppm. In the ^1^H,^13^C-meHSQC NMR spectrum its H4, *δ*_H_ 3.74, correlates to a signal at 81.26 ppm and the Δ*δ*_C4_ 8.57 for the glucuronic acid then defines the position of substitution and the residue thus corresponds to →4)-β-d-GlcA-(1→. The ^1^H and ^13^C chemical shifts of the PS–OH were correlated using the ^1^H,^13^C-meHSQC NMR spectrum and the sequence between residues, [–**B–C–D–A**–], suggested by CASPER, was confirmed by the ^1^H,^1^H-NOESY spectrum (Fig. 3b, Table 2). The predicted ^1^H and ^13^C NMR chemical shifts by CASPER were in good agreement with those assigned by the 1D and 2D NMR experiments (Fig. 6). The structure of the PS–OH is also consistent with the fact that WfdW was proposed to act via an inverting mechanism and consequently the O-antigen should contain at least one β-d-Man*p* residue, since the donor molecule used would be GDP-d-Man*p* with an α-anomeric configuration.

**Table 1:** ^1^H and ^13^C NMR chemical shifts of the O-antigen from *E. coli* O93 in which populations carrying *O*-acetyl groups are denoted by prime and double-prime residues and the corresponding *O*-deacetylated polysaccharide where sugar residues are devoid of the prime character.

Sugar residue		^1^H and ^13^C chemical shifts										
		1		2	3	4	5	6	2Ac	2CO	6Ac	6CO
**A**	→3)-α-d-GlcNAc-(1→	5.25	(M)^b^	4.06	4.06	3.61	4.21	3.83, 3.85	2.04			
		98.78	[179]^c^	53.70	81.15	69.31	72.04	61.24	22.87	N.d.^a^		
**A′**	→3)-α-d-GlcNAc-(1→	5.11		4.03	4.03	3.66	4.04	3.73, 3.91	2.03			
		99.40	{174}^d^	53.77	81.21	68.91	71.90	60.81	22.94	175.16		
**A″**	→3)-α-d-GlcNAc-(1→	5.08		4.03	4.03	3.66	4.04	3.74, 3.93	2.03			
		99.66		53.77	81.30	68.80	71.86	60.70	22.94	175.16		
**B**	→2)-β-d-Man-(1→	4.85	(S)	4.16	3.80	3.72	3.42	3.79, 3.94				
		97.55	[159]	77.33	74.37	68.04	77.54	61.78				
**B′**	→2)-β-d-Man-(1→	4.82		3.83	3.77	3.71	3.44	3.79, 3.95				
		97.22	{158}	78.02	74.28	68.09	77.46	61.80				
**B″**	→2)-β-d-Man-(1→	4.82		3.81	3.77	3.71	3.44	3.79, 3.95				
		97.17		78.41	74.18	68.09	77.41	61.89				
**C**	→3)-β-d-Man-(1→	4.62	(S)	4.17	3.85	3.62	3.39	3.75, 3.92				
		100.66	[161]	68.50	80.11	65.80	77.21	61.82				
**C′**	→3)-β-d-Man2Ac-(1→	4.81		5.53	4.04	3.54	3.47	3.77, 3.94	2.22			
		99.35	{160}	69.97	77.63	66.04	77.41	61.65	21.32	174.04		
**C″**	→3)-β-d-Man2Ac6Ac-(1→	4.82		5.55	4.07	3.61	3.67	4.37, 4.46	2.22		2.17	
		99.61		69.87	77.33	65.73	74.73	63.88	21.32	173.95	20.99	174.67
**D**	→4)-β-d-GlcA-(1→	4.59	(L)	3.40	3.65	3.74	3.81					
		103.27	[162]	73.41	74.78	81.26	76.45	N.d.^a^				
**D′**	→4)-β-d-GlcA-(1→	4.57		3.40	3.61	3.78	3.79					
		103.22	{166}	73.51	74.73	81.58	76.24	174.65				
**D″**	→4)-β-d-GlcA-(1→	4.57		3.40	3.61	3.75	3.79					
		103.22		73.44	74.73	82.14	76.24	174.65				

^a^N.d. = not determined.

^b^ ^3^ *J*_H1,H2_ value used as input for “Determine glycan structure” in CASPER: S < 2 Hz, M = 2–7 Hz and L > 7 Hz.

^c^ ^1^ *J*_C1,H1_ values given in hertz in square brackets and obtained from a coupled ^1^H,^13^C-HSQC NMR spectrum.

^d^ ^1^ *J*_C1,H1_ values given in hertz in curly brackets and calculated from a ^1^H,^13^C-CT-CE-HSQC NMR spectrum.

**Table 2:** Inter-residue correlations from ^1^H,^1^H-NOESY, 1D ^1^H,^1^H-NOESY, and ^1^H,^13^C-HMBC spectra for *E. coli* O93 O-antigen polysaccharide (PS) and de-*O*-acetylated polysaccharide (PS–OH).

Sugar residue of PS–OH			NOE	HMBC
		Atom	correlations to	via ^3^*J*_CH_
**A**	→3)-α-d-GlcNAc-(1→	H1, **A**	H2, **B**	
**B**	→2)-β-d-Man-(1→	H1, **B**	H3, **C**	
**C**	→3)-β-d-Man-(1→	H1, **C**	H4, **D**	
**D**	→4)-β-d-GlcA-(1→	H1, **D**	H3, **A**	
	PS			
**A′**	→3)-α-d-GlcNAc-(1→	H1, **A′**	H2, **B′**	C2, **B′**
		C1, **A′**		H2, **B′**
**B′**	→2)-β-d-Man-(1→	H1, **B′**	H3, **C′**	C3, **C′**
		C1, **B′**		H3, **C′**
**C′**	→3)-β-d-Man2Ac-(1→	H1, **C′**	H4, **D′**	C4, **D′**
		C1, **C′**		H4, **D′**
		Me^2Ac^, **C′**	H1, H5, H6, **A′** H1, H2, **B′**	
		Me^2Ac^, **C′**		
**D′**	→4)-β-d-GlcA-(1→	H1, **D′**	H3, **A′**	C3, **A′**
		C1, **D′**		H3, **A′**

**Fig. 7:**
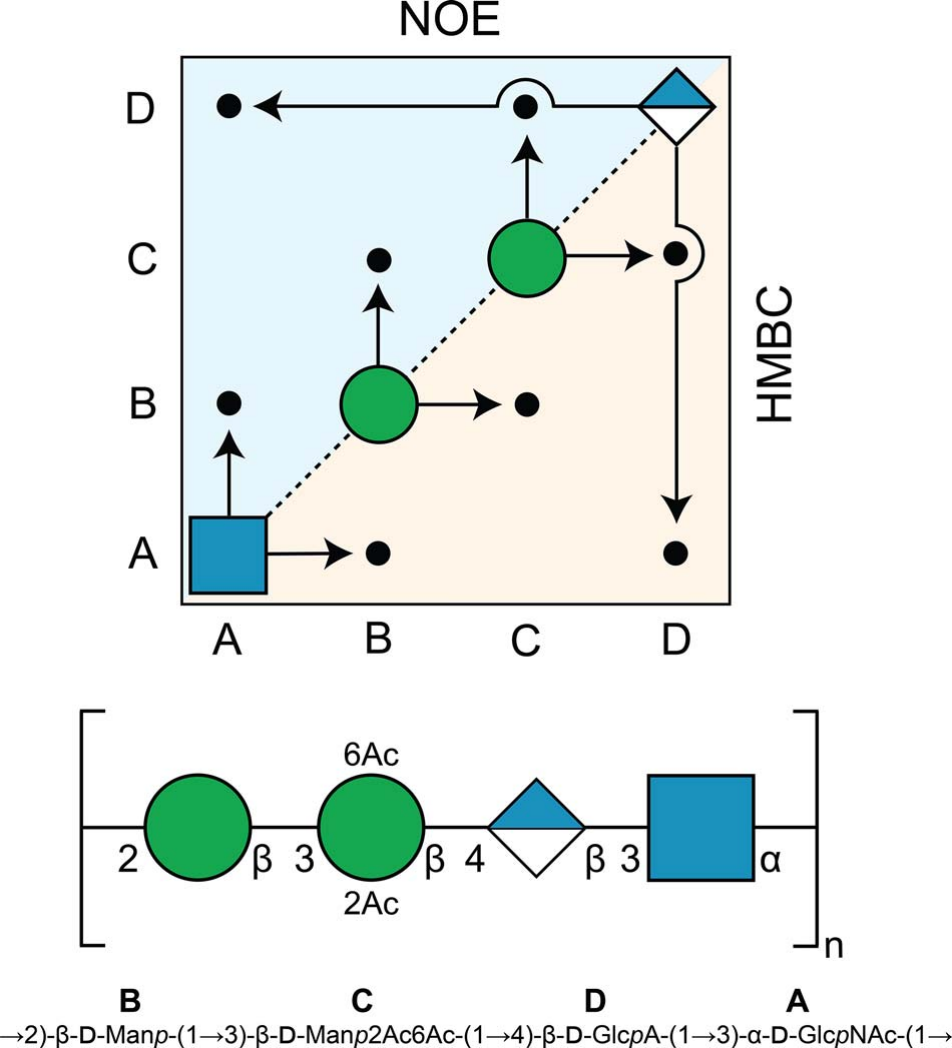
(top) A “Sequence plot” summarizing the connectivities observed in a ^1^H,^1^H-NOESY spectrum of *E. coli* O93 PS–OH (upper triangle) and a ^1^H,^13^C-HMBC spectrum of *E. coli* O93 PS (lower triangle) where the connectivities emanate “from” the anomeric position “to” the linkage position, (middle) *E. coli* O93 PS repeating unit in SNFG-representation (Neelamegham et al. 2019) and (bottom) standard nomenclature with residues annotated by **A**–**D**.

The PS of *E. coli* O93 showed ^1^H NMR resonances at 2.17 and 2.22 ppm and ^13^C NMR resonances at 20.99 and 21.32 ppm for a minor (denoted by a prime) and a major population (denoted by a double-prime), respectively, which were not present in the material after treatment under dilute basic conditions, fully consistent with the presence of *O*-acetyl groups as substituents on the sugar residue(s) of the O-antigen polysaccharide. The ^1^H,^13^C-meHSQC NMR spectrum showed characteristic chemical shift displacements as a result of *O*-acetylation, inter alia, for a 6-*O*-acetylation resulting in cross-peaks between *δ*_C_ 63.88 and the two protons at *δ*_H_ 4.37 and 4.46 (Fig. 4c), i.e. Δ*δ*_C6_ ~2 and Δ*δ*_H6_ ~0.5 (Jansson et al. 1987a). Furthermore, cross-peaks at *δ*_H_/*δ*_C_ 5.53/69.97 and *δ*_H_/*δ*_C_ 5.55/69.87 (Fig. 4d) could be assigned to the respective protons and carbons of **C2′** and **C2″** resulting in a →3)-β-d-Man2Ac-(1→ residue. The two carbonyl carbons at *δ*_C_ 174.02 and 173.95 had correlations to *δ*_H_ 5.53 and *δ*_H_ 5.55, respectively, as deduced from a ^1^H,^13^C-BS-CT-HMBC experiment (Claridge and Pérez-Victoria 2003). Interestingly, cross-peaks from *δ*_H_ 5.55 (H2 of **C″**) to *δ*_H_ 4.37 and *δ*_H_ 4.46 (H6 of **C″**) were observed in the ^1^H,^1^H-TOCSY acquired with a mixing time of 120 ms (Fig. 4e and f) showing that two *O*-acetyl groups substitute positions 2 and 6 in **C″**, i.e. a →3)-β-d-Man2Ac6Ac-(1→ residue. The ^1^H chemical shift displacements due to the *O*-acetylation at position 2 follow the pattern observed for α-l-Rha2Ac-OMe where downfield shifting by >1 ppm takes place for H2 and smaller effects in the same direction occur for H1 and H3; for the ^13^C chemical shifts the magnitude of the displacements is in the range of ~1.5–2.5 ppm, downfield for C2 and upfield for C1 and C3 (Table 1; Rönnols et al. 2013). For the PS material the anomeric configuration of, in particular, the 2 mannosyl residues was further confirmed by a ^1^H,^13^C-CT-CE-HSQC NMR experiment (Tian et al. 2001) in which 2 × ^1^*J*_C1,H1_ is determined along the *F*_1_ dimension of the spectrum (Fig. 4g, Table 1) as a way to resolve spectral overlap that may occur in an *F*_2_-coupled HSQC NMR spectrum. The degree of *O*-acetylation of residue **C** was determined by integration of the anomeric signals in the ^1^H,^13^C-HSQC NMR spectrum and showed that ~½ of the population was 2,6-di-*O*-acetylated, ~¼ was 2-*O*-acetylated, whereas ~¼ of the population did not carry *O*-acetyl groups. Whether the observed di-*O*-acetylation of a specific mannosyl residue in the RU results from the action of two different *O*-acetyl transferases, on migration (Lassfolk et al. 2019, 2022; Oh et al. 2022), or on the conditions of growth and purification of polysaccharide material (Ilg et al. 2013; Knirel et al. 2015), remains an open question.

The sequential arrangement of the sugar residues in the O93 PS was determined from ^1^H,^13^C-HMBC and ^1^H,^1^H-NOESY NMR spectra (Table 2) and is summarized in the “sequence plot” (Fig. 7), in which the correlations “from” the anomeric atoms in residues **A**–**D** “to” adjacent sugar residues are shown. To further validate the NMR resonance assignments of the PS, the suggested structure was submitted, along with the assigned ^1^H and ^13^C NMR chemicals shifts, using the “Predict NMR Chemical Shifts” entry in CASPER. The experimentally assigned and predicted NMR chemical shifts of the PS having a →3)-β-d-Man2Ac6Ac-(1→ residue as part of its RU were compared (Fig. 6) and found to be in good agreement, but for a conspicuous, but significant, ^1^H outlier for **B2″**. Significant upfield ^1^H NMR chemical shifts displacements of 0.33 and 0.35 ppm were observed for **B2′** and **B2″**, respectively, compared to **B2** in the PS–OH. Intriguingly, whereas the ^1^H NMR chemical shift of **B2** was predicted well for the PS–OH by CASPER it deviated when 2-*O*-acetyl substitution was present in residue **C**. Moreover, in residue **A**, which is not directly linked to residue **C**, the anomeric proton also showed a significant upfield chemical shift displacement of 0.14 and 0.17 ppm for **A1'** and **A1″**, respectively.

**Fig. 8:**
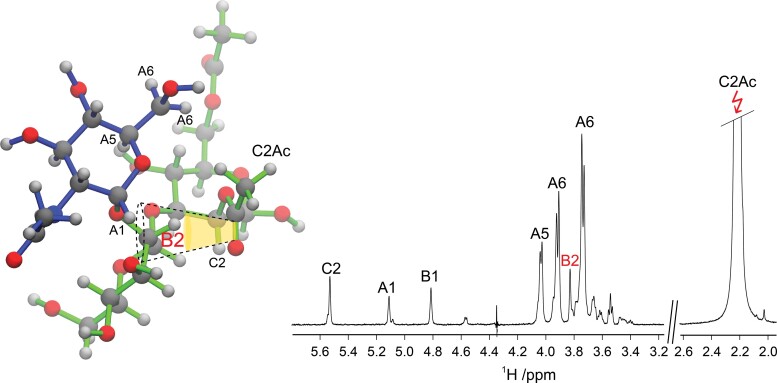
(left) 3D representation of the trisaccharide α-d-GlcNAc-(1→2)-β-d-Man-(1→3)-β-d-Man2Ac6Ac from the *E. coli* O93 PS in a conformation consistent with the 1D ^1^H,^1^H-NOESY spectrum (right) with an irradiation at 2.22 ppm of the methyl group of the *O*-acetyl group at **C2** recorded with a 600 ms mixing time at 700 MHz showing inter-residue correlations. The yellow conical shape represents the proposed anisotropic shielding effect by the carbonyl group of the *O*-acetyl group at position **C2** affecting H2 in residue **B** by an upfield chemical shift displacement, in comparison to the *O*-deacetylated polysaccharide. Primes used in the text to differentiate *O*-acetylated populations in the polysaccharide are not included in the figure for clarity.

We hypothesize that these upfield ^1^H NMR chemical shift displacements may be due to shielding by the carbonyl group, which has a strong magnetic anisotropy (Field et al. 2020) of the *O*-acetyl group in residue **C**. To further analyze this, we set out to investigate spatial proximity of protons between sugar residues and acquired 1D ^1^H,^1^H-NOESY experiments with mixing times of 300 and 600 ms where the selective irradiation was set on the methyl group of the 2-*O*-acetyl substituent (**C2″Ac**/**C2′Ac**; Fig. 8). Interestingly, strong inter-residue correlations to **A1″**, **A5″**, **A6″**, and **B2″** (and the corresponding mono-*O*-acetylated sugar residue which has degenerate or closely similar NMR chemical shifts) were observed (Fig. 8). This allowed us to construct a 3D model where **C2″Ac** was oriented in such a way that it was proximate to the protons that it had NOEs to. In this model torsion angles of ϕ_H_,ψ_H_ ≈ −35°, −45° for the glycosidic linkage of α-d-Glc*p*NAc-(1→2)-β-d-Man*p* and ϕ_H_,ψ_H_ ≈ +25°, +60° for that of β-d-Man*p*-(1→3)-β-d-Man*p*2Ac6Ac were adopted. The orientation of the carbonyl group then is such that the anisotropic shielding can affect **B2″** and the **A1″** protons, consistent with the significant upfield chemical shift displacement of these (Fig. 8).

The capsular polysaccharides (CPS) from *E. coli* K84 and *E. coli* K50 containing hexa- and pentasaccharide RUs have structural the elements →4)-β-d-Glc*p*-(1→3)-α-d-Glc*p*NAc-(1→2)-β-d-Man*p*-(1→3)-β-d-Man*p*NAc-(1→ and α-d-Glc*p*NAc-(1→2)-β-d-Man*p*4,6(*S*)Pyr-(1→3)-d-Man*p*NAc, respectively (Whittaker et al. 1994; Grue et al. 1994), which are similar to part of the O-antigen from *E. coli* O93. The ^1^H NMR chemical shifts of H1 in the α-d-Glc*p*NAc residue were 5.17 and 5.12 ppm and those for H2 in the β-d-Man*p* were 3.91 and 3.93 ppm, respectively, reminiscent of the corresponding ones in the 2-*O*-acetylated populations of the *E. coli* O93 O-antigen. Based on structural and ^1^H NMR chemical shift similarities we deduce that the presence of the *N*-acetyl group in d-Man*p*NAc of the two CPS has a similar effect on protons in adjacent sugar residues as the 2-*O*-acetyl group in d-Man*p* has on protons in the corresponding residues in the O-antigen from *E. coli* O93.

**Fig. 9:**
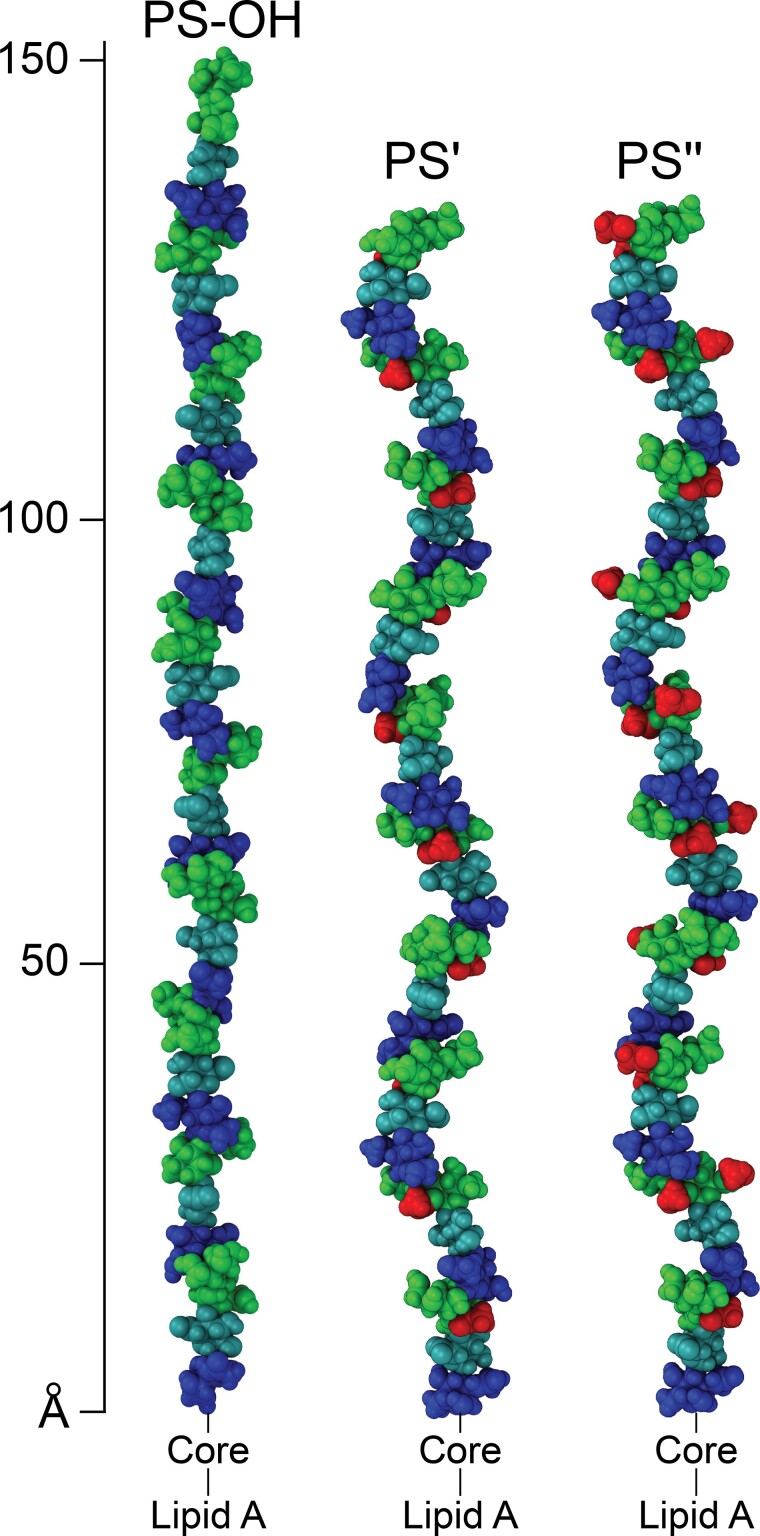
Molecular models of 10 repeating units of the *O*-deacetylated (PS–OH), 2-*O*-acetylated (PS'), and 2,6-di-*O*-acetylated (PS″) O-antigens built using CarbBuilder (Kuttel et al. 2016) and visualized with VMD (Humphrey et al. 1996). Sugar-residues are colored with d-GlcNAc in blue, d-Man in green, and d-GlcA in cyan. The *O*-acetyl groups present in (PS′) and (PS″) are depicted in red color.

From the established tetrasaccharide BRU of the *E. coli* O93 O-antigen (Fig. 7) 3D structures of the polysaccharides without and with *O*-acetyl substituents were generated by CarbBuilder (Kuttel et al. 2016; Fig. 9) revealing the *O*-acetyl groups as additional epitopes to the main chain polymer. Notably, the *E. coli* O93 O-antigen structure only differs to that of the O-polysaccharide of the gram-negative marine bacterium *Cellulophaga baltica* strain NN015840^T^ (Tomshich et al. 2007) by the additional 6-*O*-acetylation. Whereas *E. coli* O93 was grown at 37 °C, the optimal growth conditions for *C. baltica* occur at a lower temperature in the range of 26–30 °C and importantly it does not grow above 32 °C (Johansen et al. 1999). If one of the bacteria obtained the gene cluster coding for the O-antigen from the other via “horizontal gene transfer” (Liu et al. 2020; Arnold et al. 2022) remains to be elucidated.

**Fig. 10:**
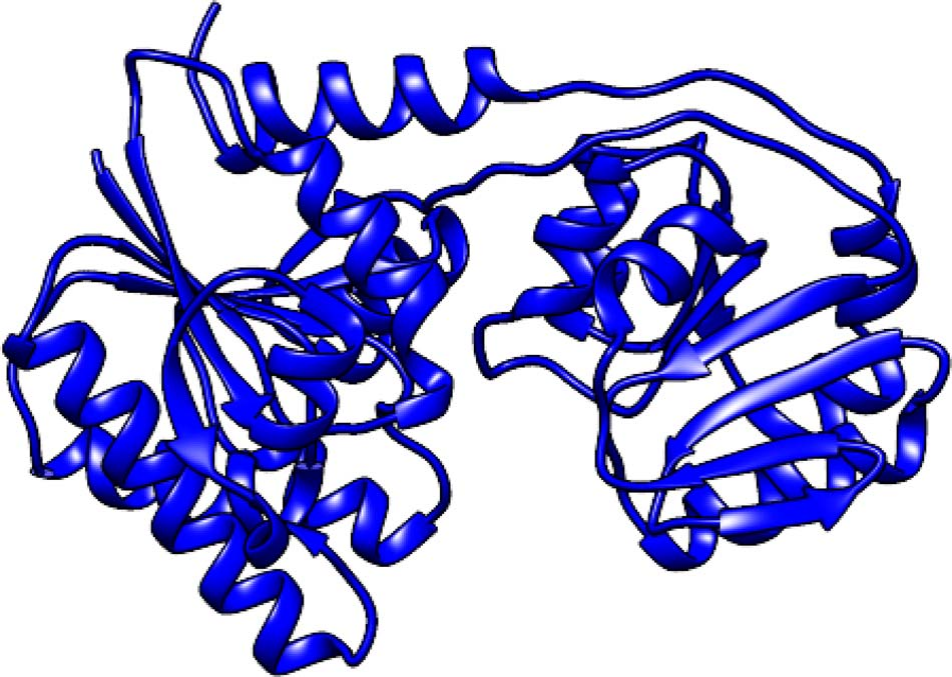
Protein structure obtained from the AlphaFold Protein Structure Database of a putative glycosyltransferase (*orf5* in the O-antigen gene cluster) from *E. coli* O93 carrying out the last glycosylation reaction in formation of the oligosaccharyl-*PP*-undecaprenyl entity. The molecular model was made using UCSF Chimera (Pettersen et al. 2004).

The analysis of function of gene products, from *galF* to *HisI* (vide supra), in relation to the determined structure of the BRU of the O-antigen from *E. coli* O93 calls for the presence of a third GT (or that WfdW has a dual activity). The function of the gene product from *orf5* was still uncharacterized and the information deposited by Iguchi et al. (2015) translated into a putative protein sequence containing 334 amino acids. A protein BLAST resulted in the identifier WP_000697476.1, which was entered to UniProt to give A0A0A8J5U3_ECOLX, followed by searching the AlphaFold Protein Structure Database (Jumper et al. 2021; Varadi et al. 2022) resulting in a hit of an uncharacterized protein, the structure of which is shown in Fig. 10. The protein has two Rossmann-like domains with βαβ-segments (Hanukoglu 2015) and displays a GT-B fold (Chang et al. 2011). Interestingly, Orf5 has a recurrent C-terminal α-helix that extends back onto the N-terminal domain, like in the GT WaaG that also has the GT-B fold (Martinez-Fleites et al. 2006; Riu et al. 2022). Moreover, whether Orf5 has a membrane-interacting region (MIR), which has been shown to present in WaaG (Liebau et al. 2015), requires further investigations. Based on the information acquired we propose that Orf5 is a GT responsible for the third sugar transfer starting from the initial GlcNAc-*PP*-undecaprenyl acceptor to result in a tetrasaccharide-*PP*-undecaprenyl entity, which subsequently is translocated from the cytoplasmic side of the inner membrane to the periplasmic side by Wzx where the oligosaccharide will be polymerized by the Wzy–Wzz complex to give the O-antigen polysaccharide.

## Conclusions

The initial analysis of the gene cluster related to the biosynthesis of the O-antigen polysaccharide from *E. coli* O93 revealed putative functions of proteins that were encoded in this part of the genome (Iguchi et al. 2015; DebRoy et al. 2016; Liu et al. 2020). This information gave clues about the biosynthesis of the O-polysaccharide, such as the presence the Wzx/Wzy biosynthetic pathway, possible constituent sugar components as well as probable *O*-acetyl decoration on the polysaccharide. Further information was obtained from ECODAB, in which the function of one of the GTs had been proposed with high fidelity, viz., the structural element β-d-Glc*p*A-(1→3)-d-Glc*p*NAc, and was consistent with the presence of UDP-glucuronic acid as the donor molecule and Und-*PP*-d-GlcNAc as the acceptor molecule in conjunction with the proposed GT family acting by an inverting mechanism.

To gain deeper insight into the structure of the O-antigen polysaccharide the bioinformatics information was coupled with an NMR analysis. Using the computer program CASPER, structure proposals were generated in a highly efficient way without the need to carry out NMR chemical shift assignments; the top-ranked proposal was subsequently confirmed to be correct by means of additional NMR experiments. Detailed NMR analysis was employed to identify the *O*-acetylation pattern of the O-antigen polysaccharide, which has tetrasaccharide RUs, →2)-β-d-Man*p*-(1→3)-β-d-Man*p*2Ac6Ac-(1→4)-β-d-Glc*p*A-(1→3)-α-d-Glc*p*NAc-(1→, consisting of three populations, viz., ~½ of them are 2,6-di-*O*-acetylated, ~¼ are 2-*O*-acetylated, and ~¼ do not carry *O*-acetyl groups on the penultimate sugar (a mannosyl residue) of the BRU. From 3D models of the different O-antigen populations it is evident that the *O*-acetyl groups can form exposed epitopes on the polysaccharide backbone, but whether the dual substitution pattern on a single sugar residue in the RU renders the bacterium any advantages as a pathogen during infection remains to be established.

## Materials and methods

### Bacterial strains and polysaccharide preparations

*Escherichia coli* reference strains of O93 (*E. coli* O93:−:H16) was obtained from SSI Diagnostica A/S (Hillerød, Denmark). Bacterial culture, LPS extraction and preparation of lipid-free polysaccharides (PS) were carried out as previously described (Furevi et al. 2020). *O*-deacetylated polysaccharide (PS–OH) was prepared by treatment of the PS with aq. NaOH (0.5 M) at 20 °C for 18 h followed by extensive dialysis against deionized water using Spectra/Por 4 Membrane Tubing with a MWCO of 12–14 kDa (fisher scientific).

### Sugar analysis by GLC

Hydrolysis of the PS was carried out using 2 M TFA at 120 °C for 30 min followed by reduction with NaBH_4_ in aq. ammonia (1 M) at ambient temp for 30 min and acetylation at 100 °C for 30 min using Ac_2_O and pyridine (1:1). The resulting mixture of alditol acetates was analyzed by gas–liquid chromatography (GLC) whereby components were separated on a PerkinElmer Elite-5 column using a temperature program of 150 °C for 2 min, 1 °C⋅min^−1^ up to 173 °C, followed by 8 °C⋅min^−1^ to 220 °C with hydrogen as the carrier gas (25 psi). The column was fitted to a PerkinElmer Clarus 400 gas chromatograph equipped with flame ionization detectors. The retention times of the derivatives were compared with those of authentic standards as references.

### NMR spectroscopy

The PS sample was deuterium-exchanged by freeze-drying from 99.9% D_2_O and examined by NMR spectroscopy at 70 °C as solutions in 99.96% D_2_O (10 mg in 0.55 mL) with a trace amount of NaN_3_ and was used for 1D ^13^C NMR, 2D ^1^H,^13^C-HMBC, ^1^H,^13^C-H2BC and ^1^H,^13^C-BS-CT-HMBC, ^1^H,^13^C-HSQC, and ^1^H,^13^C-CT-CE-HSQC experiments. A PS–OH sample purified by gel-permeation chromatography was used for further analysis employing 1D ^1^H and diffusion-filtered ^1^H NMR experiments, *F*_2_ coupled ^1^H,^13^C-HSQC, ^1^H,^13^C-HSQC, ^1^H,^1^H-TOCSY, and ^1^H,^1^H-NOESY NMR experiments (<1 mg in 0.55 mL) acquired at 60 °C. NMR spectra were recorded using a Bruker Avance III 700-MHz spectrometer equipped with 5-mm TCI (^1^H/^13^C/^15^N) Z-Gradient (53.0 G cm^−1^) CryoProbe or a Bruker Avance III 600-MHz NMR spectrometer equipped with a 5-mm TXI (^1^H/^13^C/^15^N) Z-gradient (55.7 G cm^−1^) inverse probe. Chemical shifts are reported in ppm using internal sodium 3-trimethylsilylpropanoate-2,2,3,3-*d*_4_ (TSP, *δ*_H_ 0.00) for ^1^H NMR and external 1,4-dioxane 10% in D_2_O (*δ*_C_ 67.40) for ^13^C NMR as references. Chemical shift differences were obtained by comparison with NMR data of the corresponding monosaccharides (Jansson et al. 1989). NMR experiments suitable for resonance assignments of carbohydrates (Widmalm 2021) were recorded essentially as previously described (Furevi et al. 2020); specific additional experimental conditions are given below.

^1^H,^1^H-TOCSY experiments were acquired with mixing times of 20, 30, 60, 90, and 120 for the PS–OH and 30, 60, 90, 120, and 200 ms for the PS using DIPSI-2 spin-locks. For the PS–OH, a phase-sensitive ^1^H,^1^H-NOESY experiments at 700 MHz was obtained with a mixing time of 150 ms employing homospoil gradient pulses (1 ms) during mixing time with a strength of 40% of the maximum; both types of experiments used 2,048 × 256 data points in the *F*_2_ and *F*_1_ dimensions, respectively. For the PS material, a ^1^H,^1^H-NOESY experiment, with suppression of effects from zero-quantum coherence (Thrippleton and Keeler 2003), was carried out at 600 MHz with mixing times of 60 and 150-ms employing 14 k × 256 data points in *F*_2_ and *F*_1_, respectively, an acquisition time of 1.7 s and a relaxation delay of 5 s.

Multiplicity-edited ^1^H,^13^C-HSQC experiments were acquired for PS and PS–OH at a ^1^H frequency of 700 MHz with 1,176 × 512 data points in *F*_2_ and *F*_1_, respectively, covering 100 ppm in the indirect dimension for PS and covering 105 ppm in *F*_1_ for the PS–OH. Both experiments were acquired with non-uniform sampling of 50% sparsity and an exponential weighting set to 100 ms for the *T*_2_ relaxation time. The *F*_2_ coupled ^1^H,^13^C-HSQC experiment applied to the PS–OH was recorded with 1,886 × 128 data points in the *F*_2_ and *F*_1_ dimensions, respectively, using an evolution time corresponding to ^1^*J*_CH_ of 145 Hz. A ^1^H,^13^C-CT-CE-HSQC experiment (Tian et al. 2001) was acquired for the PS with 1,176 × 512 data points in *F*_2_ and *F*_1_, respectively, and an evolution time corresponding to ^1^*J*_CH_ of 165 Hz.

A ^1^H,^13^C-HMBC experiment for the PS was acquired using a 3-fold low-pass *J*-filter, ^1^*J*_CH-min_ and ^1^*J*_CH-max_ set to 120 and 170 Hz, respectively, and a delay for the evolution of the long-range couplings corresponding to 8 Hz. The band-selective constant-time ^1^H,^13^C-HMBC experiment (Claridge and Pérez-Victoria 2003) for the PS was acquired with a delay for long-range couplings corresponding to 10 Hz, spectral widths of 8 × 6 ppm in the *F*_2_ and *F*_1_ dimensions, respectively, with the transmitter frequency at 175 ppm in the *F*_1_ dimension and a Q3 Gaussian cascade pulse for selective ^13^C excitation in the spectral region for carbonyl groups. A ^1^H,^13^C-H2BC experiment (Nyberg et al. 2005) for the PS material was recorded with a 3-fold low-pass *J*-filter, ^1^*J*_CH-min_ and ^1^*J*_CH-max_ set to 145 and 170 Hz, respectively, and a constant-time delay of 22 ms.

### Bioinformatics analysis

The bioinformatics analysis using the genome of *E. coli* O93 related to its O-antigen synthesis and expression (Iguchi et al. 2015; DebRoy et al. 2016; Liu et al. 2020) and previously deposited at NCBI (GenBank AB812041.1) by Iguchi et al. (2015) relied also on ECODAB (Lundborg et al. 2010; Rojas-Macias et al. 2015), BLAST (Camacho et al. 2009), and CAZy (Drula et al. 2022).

## Supplementary Material

SI_CASPER_inputs_O93_cwac069

## References

[ref1] Al-Dabbagh B, Olatunji S, Crouvoisier M, el Ghachi M, Blanot D, Mengin-Lecreulx D, Bouhss A. Catalytic mechanism of MraY and WecA, two paralogues of the polyprenyl-phosphate N-Acetylhexosamine 1-phosphate transferase superfamily. Biochimie. 2016:127:249–257. 27312048 10.1016/j.biochi.2016.06.005

[ref2] Arnold BJ, Huang IT, Hanage WP. Horizontal gene transfer and adaptive evolution in bacteria. Nat Rev Microbiol. 2022:20:206–218. 34773098 10.1038/s41579-021-00650-4

[ref3] Asakura H, Makino S, Shirahata T, Tsukamoto T, Kurazono H, Ikeda T, Takesh K. Detection and genetical characterization of shiga toxin-producing *Escherichia coli* from wild deer. Microbiol Immunol. 1998:42:815–822. 10037215 10.1111/j.1348-0421.1998.tb02356.x

[ref4] Beier RC, Mundy BP, Strobel GA. Assignment of anomeric configuration and identification of carbohydrate residues by 13C NMR. 1. Galacto- and glucopyranosides and furanosides. Can J Chem. 1980:58:2800–2804.

[ref5] Bettelheim KA, Kuzevski A, Gilbert RA, Krause DO, McSweeney CS. The diversity of *Escherichia coli* serotypes and biotypes in cattle faeces. J Appl Microbiol. 2005:98:699–709. 15715874 10.1111/j.1365-2672.2004.02501.x

[ref6] Bundle DR, Lemieux RU. Determination of anomeric configuration by NMR. Methods Carbohydrate Chem. 1976:7:79–86.

[ref7] Camacho C, Coulouris G, Avagyan V, Ma N, Papadopoulos J, Bealer K, Madden TL. BLAST+: architecture and applications. BMC Bioinf. 2009:10:421. 10.1186/1471-2105-10-421 PMC2803857 20003500

[ref8] Chang A, Singh S, Phillips GN, Thorson JS. Glycosyltransferase structural biology and its role in the design of catalysts for glycosylation. Curr Opin Biotechnol. 2011:22:800–808. 21592771 10.1016/j.copbio.2011.04.013 PMC3163058

[ref9] Claridge TDW, Pérez-Victoria I. Enhanced 13C resolution in semi-selective HMBC: a band-selective, constant-time HMBC for complex structure elucidation by NMR. Organic Biomol Chem. 2003:1:3632–3634. 10.1039/b307122g 14649890

[ref10] Cunneen MM, Liu B, Wang L, Reeves PR. Biosynthesis of UDP-GlcNAc, UndPP-GlcNAc and UDP-GlcNAcA involves three easily distinguished 4-epimerase enzymes, Gne, Gnu and GnaB. PLoS One. 2013:8:e67646. 23799153 10.1371/journal.pone.0067646 PMC3682973

[ref11] DebRoy C, Fratamico PM, Yan X, Baranzoni G, Liu Y, Needleman DS, Tebbs R, O’Connell CD, Allred A, Swimley M, et al. Comparison of O-antigen gene clusters of all O-serogroups of *Escherichia coli* and proposal for adopting a new nomenclature for O-typing. PLoS One. 2016:11:e0147434. 26824864 10.1371/journal.pone.0147434 PMC4732683

[ref12] Drula E, Garron ML, Dogan S, Lombard V, Henrissat B, Terrapon N. The carbohydrate-active enzyme database: functions and literature. Nucl Acids Res. 2022:50:D571–D577. 34850161 10.1093/nar/gkab1045 PMC8728194

[ref13] Fernandes JBC, Zanardo LG, Galvão NN, Carvalho IA, Nero LA, Moreira MAS. *Escherichia coli* from clinical mastitis: serotypes and virulence factors. J Vet Diagn Investig. 2011:23:1146–1152. 22362795 10.1177/1040638711425581

[ref14] Field LD, Li HL, Magill AM. 2020. Organic structures from spectra, 6th ed. John Wiley & Sons, Ltd, page 52.

[ref15] Furevi A, Ståhle J, Muheim C, Gkotzis S, Udekwu KI, Daley DO, Widmalm G. Structural analysis of the O-antigen polysaccharide from *Escherichia coli* O188. Carbohydr Res. 2020:498:108051. 33075674 10.1016/j.carres.2020.108051

[ref16] Grue MR, Parolis H, Parolis LAS. Structure of the capsular antigen of *Escherichia coli* O8:K50:H. Carbohydr Res. 1994:258:233–241. 7518740 10.1016/0008-6215(94)84089-x

[ref17] Hanukoglu I . Proteopedia: Rossmann fold: a beta-alpha-beta fold at dinucleotide binding sites. Biochem Mol Biol Educ. 2015:43:206–209. 25704928 10.1002/bmb.20849

[ref18] Humphrey W, Dalke A, Schulten K. VMD: visual molecular dynamics. J Mol Graph. 1996:14:33–38. 8744570 10.1016/0263-7855(96)00018-5

[ref19] Iguchi A, Iyoda S, Kikuchi T, Ogura Y, Katsura K, Ohnishi M, Hayashi T, Thomson NR. A complete view of the genetic diversity of the *Escherichia coli* O-antigen biosynthesis gene cluster. DNA Res. 2015:22:101–107. 25428893 10.1093/dnares/dsu043 PMC4379981

[ref20] Ilg K, Zandomeneghi G, Rugarabamu G, Meier BH, Aebi M. HR-MAS NMR reveals a pH-dependent LPS alteration by de-O-acetylation at abequose in the O-antigen of *Salmonella enterica* serovar typhimurium. Carbohydr Res. 2013:382:58–64. 24211643 10.1016/j.carres.2013.10.002

[ref21] Islam ST, Lam JS. Synthesis of bacterial polysaccharides via the Wzx/Wzy-dependent pathway. Can J Microbiol. 2014:60:697–716. 25358682 10.1139/cjm-2014-0595

[ref22] Jansson P-E, Kenne L, Schweda E. Nuclear magnetic resonance and conformational studies on monoacetylated methyl methyl D-*gluco*- and D-*galacto*-pyranosides. J Chem Soc Perkin Trans. 1987a:1:377–383.

[ref23] Jansson P-E, Kenne L, Widmalm G. CASPER-a computerised approach to structure determination of polysaccharides using information from n.m.r. spectroscopy and simple chemical analyses. Carbohydr Res. 1987b:168:67–77.

[ref24] Jansson P-E, Kenne L, Widmalm G. Computer-assisted structural analysis of polysaccharides with an extended version of casper using 1H- and 13C-n.m.r. data. Carbohydr Res. 1989:188:169–191. 2673508 10.1016/0008-6215(89)84069-8

[ref25] Jansson P-E, Kenne L, Widmalm G. Computer-assisted structural analysis of oligosaccharides using CASPER. Anal Biochem. 1991:199:11–17. 1807153 10.1016/0003-2697(91)90262-r

[ref26] Johansen JE, Nielsen P, Sjøholm C. Description of *Cellulophaga baltica* gen. nov., sp. nov. and *Cellulophaga fucicola* gen. nov., sp. nov. and reclassification of *[Cytophaga] lytica* to *Cellulophaga lytica* gen. nov., comb. nov. Int J Syst Bacteriol. 1999:49:1231–1240. 10425785 10.1099/00207713-49-3-1231

[ref27] Jumper J, Evans R, Pritzel A, Green T, Figurnov M, Ronneberger O, Tunyasuvunakool K, Bates R, Žídek A, Potapenko A, et al. Highly accurate protein structure prediction with AlphaFold. Nature. 2021:596:583–589. 34265844 10.1038/s41586-021-03819-2 PMC8371605

[ref28] Kabeya H, Sato S, Oda S, Kawamura M, Nagasaka M, Kuranaga M, Yokoyama E, Hirai S, Iguchi A, Ishihara T, et al. Characterization of shiga toxin-producing *Escherichia coli* from feces of sika deer (*Cervus nippon*) in Japan using PCR binary typing analysis to evaluate their potential human pathogenicity. J Vet Med Sci. 2017:79:834–841. 28320988 10.1292/jvms.16-0568 PMC5447969

[ref29] Klein G, Müller-Loennies S, Lindner B, Kobylak N, Brade H, Raina S. Molecular and structural basis of inner core lipopolysaccharide alterations in *Escherichia coli*: incorporation of glucuronic acid and phosphoethanolamine in the heptose region. J Biol Chem. 2013:288:8111–8127. 23372159 10.1074/jbc.M112.445981 PMC3605630

[ref30] Knirel YA, Sun Q, Senchenkova SN, Perepelov A, v., Shashkov AS, Xu J. O-Antigen modifications providing antigenic diversity of *Shigella flexneri* and underlying genetic mechanisms. Biochem Mosc. 2015:80:901–914. 10.1134/S0006297915070093 26542003

[ref31] Kuttel MM, Ståhle J, Widmalm G. CarbBuilder: software for building molecular models of complex oligo- and polysaccharide structures. J Comput Chem. 2016:37:2098–2105. 27317625 10.1002/jcc.24428

[ref32] Lassfolk R, Rahkila J, Johansson MP, Ekholm FS, Wärnå J, Leino R. Acetyl group migration across the saccharide units in oligomannoside model compound. J Am Chem Soc. 2019:141:1646–1654. 30586298 10.1021/jacs.8b11563

[ref33] Lassfolk R, Pedrón M, Tejero T, Merino P, Wärnå J, Leino R. Acyl group migration in pyranosides as studied by experimental and computational methods. Chemistry—A Eur J. 2022:28:e202200499. 10.1002/chem.202200499 PMC9322027 35302249

[ref34] Li X, Perepelov AV, Wang Q, Senchenkova SN, Liu B, Shevelev SD, Guo X, Shashkov AS, Chen W, Wang L, et al. Structural and genetic characterization of the O-antigen of *Escherichia coli* O161 containing a derivative of a higher acidic diamino sugar, legionaminic acid. Carbohydr Res. 2010:345:1581–1587. 20510395 10.1016/j.carres.2010.04.008

[ref35] Liebau J, Pettersson P, Szpryngiel S, Mäler L. Membrane interaction of the glycosyltransferase WaaG. Biophys J. 2015:109:552–563. 26244737 10.1016/j.bpj.2015.06.036 PMC4571021

[ref36] Liu MA, Morris P, Reeves PR. Wzx flippases exhibiting complex O-unit preferences require a new model for Wzx–substrate interactions. MicrobiologyOpen. 2019:8:e655. 10.1002/mbo3.655 PMC6436433 29888516

[ref37] Liu B, Furevi A, Perepelov AV, Guo X, Cao H, Wang Q, Reeves PR, Knirel YA, Wang L, Widmalm G. Structure and genetics of *Escherichia coli* O antigens. FEMS Microbiol Rev. 2020:44:655–683. 31778182 10.1093/femsre/fuz028 PMC7685785

[ref38] Lo Leggio L, Dal Degan F, Poulsen P, Andersen SM, Larsen S. The structure and specificity of *Escherichia coli* maltose acetyltransferase give new insight into the lacA family of acyltransferases. Biochemistry. 2003:42:5225–5235. 12731863 10.1021/bi0271446

[ref39] Lundborg M, Widmalm G. Structural analysis of glycans by NMR chemical shift prediction. Anal Chem. 2011:83:1514–1517. 21280662 10.1021/ac1032534

[ref40] Lundborg M, Modhukur V, Widmalm G. Glycosyltransferase functions of *E. coli* O-antigens. Glycobiology. 2010:20:366–368. 19926726 10.1093/glycob/cwp185

[ref41] Martinez-Fleites C, Proctor M, Roberts S, Bolam DN, Gilbert HJ, Davies GJ. Insights into the synthesis of lipopolysaccharide and antibiotics through the structures of two retaining glycosyltransferases from family GT4. Chem Biol. 2006:13:1143–1152. 17113996 10.1016/j.chembiol.2006.09.005

[ref42] Melton-Celsa AR . Shiga toxin (Stx) classification, structure, and function. 2014: *Microbiology Spectrum* 2: EHEC-0024-2013. 10.1128/microbiolspec.EHEC-0024-2013 PMC4270005 25530917

[ref43] Morita-Ishihara T, Iyoda S, Iguchi A, Ohnishi M. 2016. Secondary shiga toxin–producing *Escherichia coli* infection, Japan, 2010–2012. Emerg Infect Dis 22: 2181–2184. 27869602 10.3201/eid2212.160783 PMC5189154

[ref44] Neelamegham S, Aoki-Kinoshita K, Bolton E, Frank M, Lisacek F, Lütteke T, O’Boyle N, Packer NH, Stanley P, Toukach P, et al. Updates to the symbol nomenclature for glycans guidelines. Glycobiology. 2019:29:620–624. 31184695 10.1093/glycob/cwz045 PMC7335484

[ref45] Nyberg NT, Duus JØ, Sørensen OW. Heteronuclear two-bond correlation: suppressing heteronuclear three-bond or higher NMR correlations while enhancing two-bond correlations even for vanishing 2JCH. J Am Chem Soc. 2005:127:6154–6155. 15853304 10.1021/ja050878w

[ref46] Oh L, Ji Y, Li W, Varki A, Chen X, Wang L-P. *O*-acetyl migration within the sialic acid side chain: a mechanistic study using the *ab initio* nanoreactor. Biochemistry. 2022:61:2007–2013. 36054099 10.1021/acs.biochem.2c00343 PMC9588306

[ref47] Pelayo JS, Elias Junior AR, de Lima NR, Navarro A, da Rocha SPD. Detection of diarrheagenic *Escherichia coli* in bovine meat in the northern region of Parana state, Brazil. Braz Arch Biol Technol. 2019:62:e19180012.

[ref48] Pettersen EF, Goddard TD, Huang CC, Couch GS, Greenblatt DM, Meng EC, Ferrin TE. UCSF Chimera – a visualization system for exploratory research and analysis. J Comput Chem. 2004:25:1605–1612. 15264254 10.1002/jcc.20084

[ref49] Reeves PR, Hobbs M, Valvano MA, Skurnik M, Whitfield C, Coplin D, Kido N, Klena J, Maskell D, Raetz CRH, et al. Bacterial polysaccharide synthesis and gene nomenclature. Trends Microbiol. 1996:4:495–503. 9004408 10.1016/s0966-842x(97)82912-5

[ref50] Ritchie RGS, Cyr N, Korsch B, Koch HJ, Perlin AS. Carbon-13 chemical shifts of furanosides and cyclopentanols. Configurational and conformational influences. Can J Chem. 1975:53:1424–1433.

[ref51] Riu F, Ruda A, Engström O, Muheim C, Mobarak H, Ståhle J, Kosma P, Carta A, Daley DO, Widmalm G. A lead-based fragment library screening of the glycosyltransferase WaaG from *Escherichia coli*. Pharmaceuticals. 2022:15:209. 35215321 10.3390/ph15020209 PMC8877264

[ref52] Rojas-Macias MA, Ståhle J, Lütteke T, Widmalm G. Development of the ECODAB into a relational database for *Escherichia coli* O-antigens and other bacterial polysaccharides. Glycobiology. 2015:25:341–347. 25352573 10.1093/glycob/cwu116

[ref53] Rönnols J, Pendrill R, Fontana C, Hamark C, Angles, d’Ortoli T, Engström O, Ståhle J, Zaccheus M v, Säwén E, Hahn LE, et al. Complete 1H and 13C NMR chemical shift assignments of mono- to tetrasaccharides as basis for NMR chemical shift predictions of oligosaccharides using the computer program CASPER. Carbohydr Res. 2013:380:156–166. 24036391 10.1016/j.carres.2013.06.026

[ref54] Ståhle J, Widmalm G. Lipopolysaccharides of gram-negative bacteria: biosynthesis and structural aspects. Trends Glycosci Glycotechnol. 2019:31:E159–E171.

[ref55] Stenutz R, Weintraub A, Widmalm G. The structures of *Escherichia coli* O-polysaccharide antigens. FEMS Microbiol Rev. 2006:30:382–403. 16594963 10.1111/j.1574-6976.2006.00016.x

[ref56] Stevenson G, Andrianopoulos K, Hobbs M, Reeves PR. Organization of the *Escherichia coli* K-12 gene cluster responsible for production of the extracellular polysaccharide colanic acid. J Bacteriol. 1996:178:4885–4893. 8759852 10.1128/jb.178.16.4885-4893.1996 PMC178271

[ref57] Thrippleton MJ, Keeler J. Elimination of zero-quantum interference in two-dimensional NMR spectra. Ang Chem - Int Ed. 2003:42:3938–3941. 10.1002/anie.200351947 12949874

[ref58] Thu HTV, Anh DTL, Dong L v. *Escherichia coli* infection in ducks in the Mekong Delta: Bacterial isolation, serogroup distribution and antibiotic resistance. Can Tho Univ J Sci. 2019:11:24–29.

[ref59] Tian F, Al-Hashimi HM, Craighead JL, Prestegard JH. Conformational analysis of a flexible oligosaccharide using residual dipolar couplings. J Am Chem Soc. 2001:123:485–492. 11456551 10.1021/ja002900l

[ref60] Tomshich SV, Komandrova NA, Widmalm G, Nedashkovskaya OI, Shashkov AS, Perepelov AV. Structure of acidic O-specific polysaccharide from the marine bacterium *Cellulophaga baltica*. Russian J Bioorg Chem. 2007:33:83–87. 10.1134/s1068162007010104 17375664

[ref61] Varadi M, Anyango S, Deshpande M, Nair S, Natassia C, Yordanova G, Yuan D, Stroe O, Wood G, Laydon A, et al. AlphaFold Protein Structure Database: massively expanding the structural coverage of protein-sequence space with high-accuracy models. Nucl Acids Res. 2022:50:D439–D444. 34791371 10.1093/nar/gkab1061 PMC8728224

[ref62] Vila J, Sáez-López E, Johnson JR, Römling U, Dobrindt U, Cantón R, Giske CG, Naas T, Carattoli A, Martínez-Medina M, et al. *Escherichia coli*: an old friend with new tidings. FEMS Microbiol Rev. 2016:40:437–463. 28201713 10.1093/femsre/fuw005

[ref63] Wang Y, Tang C, Yu X, Xia M, Yue H. Distribution of serotypes and virulence-associated genes in pathogenic *Escherichia coli* isolated from ducks. Avian Pathology. 2010:39:297–302. 20706886 10.1080/03079457.2010.495742

[ref64] Whitfield C, Williams DM, Kelly SD. Lipopolysaccharide O-antigens-bacterial glycans made to measure. J Biol Chem. 2020:295:10593–10609. 32424042 10.1074/jbc.REV120.009402 PMC7397119

[ref65] Whittaker DV, LAS P, Parolis H. Structural elucidation of the capsular polysaccharide produced by *E. coli* O20:K84:H26. Carbohydr Res. 1994:262:323–334. 7982223 10.1016/0008-6215(94)84188-8

[ref66] Widmalm G . General NMR spectroscopy of carbohydrates and conformational analysis in solution. In: Barchi JJ Jr, editors. Comprehensive glycoscience. 2nd ed. Elsevier Inc.; 2021. pp. 340–373

[ref67] Yethon JA, Vinogradov E, Perry MB, Whitfield C. Mutation of the lipopolysaccharide core glycosyltransferase encoded by waaG destabilizes the outer membrane of *Escherichia coli* by interfering with core phosphorylation. J Bacteriol. 2000:182:5620–5623. 10986272 10.1128/jb.182.19.5620-5623.2000 PMC111012

